# Photon strength functions and nuclear level densities: invaluable input for nucleosynthesis

**DOI:** 10.1098/rsta.2023.0125

**Published:** 2024-07-23

**Authors:** M. Wiedeking, S. Goriely

**Affiliations:** ^1^ SSC Laboratory, iThemba LABS, P.O. Box 722, Somerset West 7129, South Africa; ^2^ School of Physics, University of the Witwatersrand, Johannesburg 2050, South Africa; ^3^ Nuclear Science Division, Lawrence Berkeley National Laboratory, Berkeley, CA 94720, USA; ^4^ Institut d’Astronomie et d’Astrophysique, Université Libre de Bruxelles, Campus de la Plaine CP 226, Brussels 1050, Belgium

**Keywords:** photon strength function, nuclear level density, reaction rates, i-process, r-process, p-process

## Abstract

The pivotal role of nuclear physics in nucleosynthesis processes is being investigated, in particular the intricate influence of photon strength functions (PSFs) and nuclear level densities (NLDs) on shaping the outcomes of the i-, r- and p-processes. Exploring diverse NLD and PSF model combinations uncovers large uncertainties for (p, γ ), (n, γ ) and ( α , γ ) rates across many regions of the nuclear chart. These lead to potentially significant abundance variations of the nucleosynthesis processes and highlight the importance of accurate experimental nuclear data. Theoretical insights and advanced experimental techniques lay the ground work for profound understanding that can be gained of nucleosynthesis mechanisms and the origin of the elements. Recent results further underscore the effect of PSF and NLD data and its contribution to understanding abundance distributions and refining knowledge of the intricate nucleosynthesis processes.

This article is part of the theme issue ‘The liminal position of Nuclear Physics: from hadrons to neutron stars’.

## 1. Introduction

Nuclear physics lies at the heart of understanding the fundamental constituents and interactions that govern the synthesis of matter in the universe. As we strive to solve the mysteries of the cosmos, nuclear structure and reaction properties play a pivotal role in shaping the evolution of stars, the synthesis of elements and the dynamics of stellar explosions. Crucial to unravelling these astrophysical phenomena is a comprehensive understanding of the underlying nuclear properties, particularly nuclear level densities (NLDs) and photon strength functions (PSFs).

PSFs and NLDs are fundamental quantities encapsulating crucial information about nuclear structure and decay mechanisms and are key parameters that significantly influence the outcomes of various nuclear reaction processes. The PSF quantifies the average probability of γ -ray absorption or emission as a function of γ -ray energy. The NLD on the other hand characterizes the distribution of nuclear states as a function of angular momentum, parity and excitation energy, hence influencing cross sections and reaction rates. For nuclear astrophysics, these quantities are indispensable in deciphering the nucleosynthesis mechanisms and interpreting the observed elemental abundances in cosmic objects. PSFs and NLDs underlie the production and destruction of nuclei in different astrophysical environments. Whether it is the rapid, intermediate or slow neutron-capture processes (r-, i- and s-processes) or the photo-disintegration process (p-process) responsible for creating heavy elements, these input parameters influence the ultimate outcome of nucleosynthesis abundance distributions. Advancements in experimental techniques, theoretical models and computational capabilities have led to remarkable progress in the determination and understanding of nuclear structure and reactions, as well as the associated creation of nuclei in the cosmos.

The accurate determination of NLDs and PSFs constitutes a significant challenge in experimental nuclear physics research. Over the last decade, incredible progress has been made in developing experimental and analytical techniques that allow greater accessibility to experimentally study nuclei even away from stability. Recent measurements have provided new constraints to theoretical models that have been shifting in the last decade from a phenomenological to a microscopic description of the nucleus and of its NLD and PSF properties.

This article aims to present an overview of the experimental and theoretical advances to understand PSFs and NLDs and their indispensability in nuclear astrophysics. By reviewing recent experimental progress, theoretical developments and their implications on our understanding of nucleosynthesis in the cosmos, this work seeks to highlight the pivotal role played by these input parameters in unravelling the origin of the elements in the Universe.

## 2. PSFs

### (a) Experimental methods

Information on the electromagnetic dipole response of a nucleus can be obtained through a large range of experimental techniques [1], including photo-nuclear reactions, nuclear resonance fluorescence (NRF), two-step cascade, primary transitions measured in inelastic proton scattering with a polarized beam, neutron- and proton-capture and charged particle reactions.

Above the neutron separation energy $S_{n}$ , spanning the energy range of the giant dipole resonance (GDR), the dipole PSFs can be obtained from photo-neutron cross sections, which are measured as a function of the photon energy. This is achieved through monochromatic γ -ray beams produced mostly by positron annihilation-in-flight and through bremsstrahlung beams [2]. Partial photo-neutron reaction cross sections are typically determined by directly detecting neutrons and quantifying the residual γ -ray activity through counting. Additional measurements with protons in the outbound reaction channels are utilized for the determination of the total photo-nuclear cross section.

At $S_{n}$ , data may be available from neutron resonance capture with white neutron beams or time-of-flight techniques. Discrete resonance capture averages isolated resonances data, while for the average resonance capture, the averaging is an intrinsic feature of the neutron beam energy range. From these, information on the PSF is generally available for γ -ray energies from $S_{n}$ and 2 to 3 MeV below $S_{n}$ [3,4]. On the other hand, information on the PSF can be extracted from thermal or cold neutron captures where the relative intensities of primary γ -rays per captures can be converted to partial radiative widths to individual final levels [5].

Below $S_{n}$ , information on the dipole PSF can be obtained for instance through photon scattering in NRF measurements, where nuclear states are excited from the ground state via the absorption of mostly dipole photons. NRF experiments determine the photo-absorption cross section on an absolute scale. Experiments either use quasi-monoenergetic photons as provided for example by the high-intensity γ -ray source [6] or broad-band bremsstrahlung from the γ ELBE facility [7]. High-purity germanium (HPGe) detectors are typically used to measure the γ -ray intensities from transitions of interest depending on the analytical method used, as summarized in Ref. [1].

The Oslo method, on the other hand, enables the simultaneous extraction of the PSF and NLD from particle- γ coincidence data below $S_{n}$ [8]. Experimentally, particle telescopes measure the energies of outgoing charged particles. Arrays of NaI(Tl), LaBr3:Ce or HPGe detectors measure the emitted γ -rays. These particle- γ coincidence data are organized into a matrix of excitation energy ( $E_{x}$ ) and γ -ray energy ( $E_{\gamma }$ ).

The first step of the analysis is to unfold the γ -ray spectra [9], utilizing customized response functions for the specific characteristics of the γ -ray detectors used. An iterative subtraction technique which is known as the first-generation method [10] extracts the distribution of primary γ -rays. The method is based on the assumption of the Brink–Axel hypothesis [11,12], positing the independence of γ -decay from a specific excitation energy origin, whether via direct population from reactions or through γ -ray decay emanating from higher-lying states.

At the core of the analysis lies the intrinsic relationship between the NLD, denoted as $\rho \,\left(E_{f}\right)$ at the final excitation energy $E_{f}=E_{x}-E_{\gamma }$ , and the total γ -ray transmission coefficient ${𝒯}_{\gamma }\,\left(E_{\gamma }\right)$ with dipole transitions being dominant [13,14]. The interplay between the level density and the γ -transmission coefficient, representing the primary γ -ray spectrum, $P\,\left(E_{\gamma },E_{x}\right)$ , is expressed as


(2.1) $$P\,\left(E_{\gamma },E_{x}\right)\propto \rho \,\left(E_{f}\right)\,{𝒯}_{\gamma }\,\left(E_{\gamma }\right).$$

The extraction process entails a $\chi^{2}$ fit [8] to derive the functional forms of $\rho \,\left(E_{f}\right)$ and ${𝒯}_{\gamma }\,\left(E_{\gamma }\right)$ . The procedure is confined to the specific energy range of the primary γ -ray matrix where statistical decay dominates.

The normalization of the transmission coefficients is achieved with the average total radiative width $\Gamma_{\gamma }$ of neutron resonances [15], facilitating their conversion into the absolute value of the PSF, which encompasses both E⁢1 and M⁢1 contributions. In cases where $\Gamma_{\gamma }$ is not readily available, systematics can be employed as substitutes from suitable mass regions, neighbouring nuclei or models, refer e.g [16–18]. The functional form of the PSF is closely linked to the slope of the NLD which will be discussed in §2b.

Recent advancements have expanded the frontiers of the Oslo method to allow investigations of the PSFs in neutron-rich nuclei or for nuclei at or near stability where chemical or physical properties of isotopes make manufacturing targets challenging. This is achieved through the analysis of γ -ray data in total absorption spectroscopy following beta-decay, known as the beta-Oslo method [19], or through the utilization of inverse kinematic reactions with stable [20] and radioactive ion beams [16]. Importantly, these innovative experimental approaches all utilize the construction of $E_{x}$ versus $E_{\gamma }$ matrices, forming the foundation for the application of the Oslo method despite the varying excitation-energy resolutions for the different methods with full widths at half maximum ranging from approximately ± 0.07 to 1 MeV. Normalizations for Oslo-type measurements rely on discrete levels at low excitation energies, the neutron resonance spacings and radiative widths at $S_{n}$ . Neutron resonance data may be unavailable in particular for nuclei away from stability which leads to an increase in the NLD and PSF uncertainties. In contrast to resonance information, the low-lying level structure will probably be known and can be utilized to place constraints on the slope of the PSF and NLD [21,22]. In the absence of average radiative widths, Coulomb dissociation experiments can provide constraints on the absolute value of the PSF for unstable nuclei [16].

Inelastic proton scattering with high-energy polarized beams bridges the energy region across $S_{n}$ providing data up to $E_{x}∼$ 20 MeV [23]. The protons being measured with magnetic spectrometers at forward angles ensures that virtual Coulomb excitation dominates and the analytical method allows for a separation of E⁢1 and M⁢1 contributions to the PSF.

PSF measurements are a suitable tool to explore the evolution of resonances. For instance, the E⁢1 pygmy dipole resonance (PDR) is located in the vicinity of $S_{n}$ for nuclei with an excess of neutrons. The PDR is often explored with isoscalar, e.g. ( α , α ′) at 120 MeV, and isovector, e.g. ( γ , γ ′), probes which reveal a separation into isoscalar and isovector excitations. The PDR is also populated in lower-energy charged-particle reactions as demonstrated for ^74^Ge [24] or through Oslo method measurements [25,26] although the latter does not generally allow for an unambiguous identification as a E⁢1 PDR.

The M⁢1 scissors mode strength is generally found to be concentrated at $E_{\gamma }∼3$ MeV as measured in several deformed nuclei with Oslo- [26,27] and NRF-type [28] experiments. For the heaviest nuclei, some PSFs have revealed a splitting of the scissors mode from the Oslo method [17,29,30] and NRF experiments [31,32]. This splitting has been proposed to be either a measure of triaxiality [33–35] or owing to the spin scissors component ([36] and references therein).

The Oslo method has also unexpectedly shown an increase in the PSF for $E_{\gamma }\to 0$ MeV [37 *].* Initially observed in *A* < 100 nuclei, e.g. [18,25], it has recently been observed in some heavier nuclei as well [14,26,38]. Since its discovery, this so-called ‘upbend’ has been independently confirmed [39] and shown to have dipole multipolarity [14] and magnetic polarization although not conclusively [40]. The experimental discovery of the upbend which was not theoretically predicted has consequently triggered new theoretical developments.

### (b) Theoretical models

Basic semi-classical models posit a Lorentzian shape for the photo-absorption cross section primarily governed by the GDR, especially in the case of medium- and heavy-mass nuclei. Substantial deviations from a conventional Lorentzian (SLO) have been observed below $S_{n}$ in experimental and theoretical studies [13]. A generalization of the Lorentzian shape, for data below and above the neutron threshold, was accomplished by incorporating a width that depends on energy and temperature. This set of Lorentzian models is grounded in the theory of Fermi liquids [41] and has demonstrated considerable improvements in calculations of the experimental radiative widths and γ -ray spectra. Until recently, the generalized Lorentzian functional has been the exclusive model for the E⁢1 mode in practical applications, especially in global calculations involving large sets of nuclei. Despite the widespread study of the E⁢1 mode, comparatively less attention has been given to parametrize the M⁢1 PSF. The common formula for the M1 PSF is an SLO expression characterizing only the spin-flip mode [13,42] neglecting the M⁢1 scissors mode in deformed nuclei. Only a limited number of works [43,44] propose a systematic phenomenological description.

The Lorentzian GDR approach, however, even in the generalized form, suffers from shortcomings of various sorts. On the one hand, it is unable to predict the enhancement of the E⁢1 strength at energies around the neutron separation energy (such as the pygmy resonance) as demonstrated by different experiments. On the other hand, even if a Lorentzian function provides a suitable representation of the E⁢1 strength, the location of its maximum and its width remain to be predicted from a model for each nucleus or from systematics.

In view of this, and considering the potential substantial effect of the GDR properties and low-energy resonances on the predictions of the radiative capture cross sections, microscopic PSF models have been developed with the aim of greater reliability and predictive power. Since the early 1970s, various mean-field approaches, including the quasi-particle random phase approximation (QRPA), the quasiparticle-phonon model and their improved variants, have been developed and successfully applied to describe giant multipole resonances in both non-relativistic and relativistic frameworks. The nuclear shell model has also been extensively utilized to describe electromagnetic excitations albeit restricted to light nuclei. Despite significant development of microscopic models, only a few attempts have been made to provide systematic large-scale calculations of both the E⁢1 and M⁢1 modes that can compete with more phenomenological Lorentz-type models. QRPA PSFs obtained within the non-relativistic Hartree–Fock–Bogoliubov (HFB) [45–48] frameworks have demonstrated a satisfactory reproduction of the GDR’s location and width, as well as the average resonance capture data at low energies, covering a wide range of existing data. The aforesaid QRPA calculations have been performed for all 8≤Z≤110 nuclei between the two drip lines. Similar attempts within the relativistic mean-field framework have been made in a less systematic manner [49,50] focusing only on the E⁢1 mode.

When considering the de-excitation PSF, variations from the photo-absorption strength can be anticipated, particularly as γ -ray energies approach the zero limit. As previously mentioned, experimental confirmation of a low-energy enhancement in the de-excitation PSF has been established. Self-consistent temperature-dependent QRPA calculations have been proposed to elucidate the low-energy upbend, suggesting an E⁢1 character [51]. Shell model calculations indicate a potential M⁢1 origin for this phenomenon [52]. Intriguingly, shell model results also suggest a connection between the upbend and scissors resonance [53,54]. The existence of the upbend has prompted new theoretical and experimental investigations into low-lying M⁢1 strength and its influence on astrophysical reaction rates. This effect has been demonstrated to be significant, leading to an increase in the rates up to a factor of 50–100 close to the neutron drip line [44,47,55].

## 3. NLDs

### (a) Experimental methods

Experimentally, several methods exist to obtain information on the NLD of a nucleus. Some techniques probe spins and parities very selectively with magnetic spectrometers and a wavelet analysis of the discrete wavelet transform [56]. Another technique to obtain partial NLDs utilizes neutron time-of-flight experiments to measure neutron resonances at $S_{n}$ , which typically provide ℓ=0 neutron resonance spacing ( $D_{0}$ ) and occasionally ℓ=1 neutron resonance spacing ( $D_{1}$ ) giving limited information on the NLD at a very narrow $E_{x}$ and spin interval ( ℓ±1/2 ) [42,57]. Such measurements are generally only available for nuclei ( A + 1) in the immediate vicinity of a stable isotope ( A ).

NLDs can also be obtained from particle-evaporation spectra of the compound nucleus, e.g. [58]. It is important to only extract data that originate from a compound nucleus and discard other reaction channels. From such measurements, the NLD at high $E_{x}$ populates a large spin distribution in the compound and final nuclei. The data are then extrapolated to the low-energy regime where the NLD is available from the number of discrete levels. The analysis does not require the normalization parameter $D_{0}$ but is model-dependent as it utilizes the Hauser–Feshbach model [59], which predicts the cross section of evaporation channels, from which the measured evaporation spectrum is being determined by the total NLD.

For the Oslo method, the normalization of the NLD is achieved at low excitation energies by comparing the data with discrete levels. The determination of the total NLD at $S_{n}$ is accomplished through the spin cutoff parameter and the average neutron resonance spacing $D_{0}$ [8,60]. The NLD is extrapolated to $S_{n}$ using empirical NLD models such as for instance the constant temperature [61], back-shifted Fermi gas [62] or microscopically with the HFB plus combinatorial model (HFB+comb) [63], from the highest excitation energy accessible through the Oslo method. However, the normalization of Oslo method NLD data with $D_{0}$ , even if accurately known, remains non-trivial, as discussed in Ref. [64]. In particular, large uncertainties still affect the spin cutoff factor and more generally the NLD spin distribution which can noticeably differ from the statistical Gaussian limit with an energy-independent moment of inertia (refer to [42,65] for more details). In cases where $D_{0}$ is not readily available, systematics or models are used, e.g. [16–18].

Since information on $D_{0}$ data is restricted to $S_{n}$ with a narrow spin range and for nuclei in the immediate vicinity of the line of stability, their reliability to normalize the results of the Oslo method can be limited. The absence of measured $D_{0}$ values poses challenges. In this context, the shape method has been developed as a versatile prescription applicable to a wide range of nuclides [66]. This method leverages the unambiguous identification of excitation energy and its subsequent direct feeding of low-lying levels. Primary transitions from the same excitation energy region to different discrete levels carry vital information regarding the functional form of the PSF and, consequently, the slope of the NLD. The shape method draws from concepts of the average resonance proton capture [1], and the ratio and $\chi^{2}$ methods, which have been developed [39], tested [67] and applied [40] over the last decade.

A comparative analysis between the Oslo and shape methods reveals that they complement each other in terms of strengths and limitations. The Oslo method excels at the lowest γ -ray energies but may encounter challenges at higher energies. Conversely, the shape method continues to exhibit robustness at the highest γ -ray energies but Porter Thomas (PT) fluctuations [68] may influence results at low γ -ray energies, particularly in light or close-to-spherical nuclei with low NLDs. Investigations into the influence of PT fluctuations on the PSFs and NLDs in ^120,124^Sn isotopes have been carried out [69] and suggest that the lowest energies may be dominated by PT fluctuations or other nuclear structure effects.

Recent applications of the shape method in conjunction with the beta-Oslo Method for ^76^Ge and ^88^Kr, measured in radioactive ion beam experiments, demonstrate the potential in constraining the slope of the NLD and showcase the shape method’s efficacy [22]. It is worthwhile to point out that, despite the narrow spin range of populated levels in β decay, the NLDs can be reliably obtained as shown for ^51^Ti which was populated in both, a charged-particle reaction and following β decay [70]. To date, the shape method has been used on several other nuclei such as ^93^Sr [71], various Nd isotopes [26] and ^97,100^Mo [72], expanding the breadth of the method reach and contributing to the understanding of the general applicability of the shape method and potential limits which may be encountered should a clear identification of low-lying states be difficult as may be the case for odd–odd nuclei.

### (b) Theoretical models

NLDs have been a subject of extensive theoretical investigation going back as early as 1936, marked by Bethe’s groundbreaking work [73]. The widely used method for calculating NLDs is the so-called ‘partition function method’, primarily owing to its ability to provide simple analytical formulae. In its most basic form, the NLD is evaluated for a gas of non-interacting fermions confined within the nuclear volume with equally spaced energy levels. Such a model corresponds to the zeroth order approximation of a Fermi gas model, resulting in analytical expressions that, while simple, are deemed unreliable. Numerous phenomenological adjustments to Bethe’s original analytical formulation have been proposed, especially to account for shell, pairing, deformation and collective effects. This gave rise initially to the constant temperature formula, followed by the shifted Fermi gas model, and subsequently, the widely used back-shifted Fermi gas model (refer, e.g. Refs. [42,74] and references therein).

Level densities play a key role for modelling nuclear reactions. As new facilities and innovative experiments are developed, and considering the need for astrophysical applications, there is a demand for nuclear data beyond the valley of stability. This poses challenges for NLD models. Indeed, cross-section predictions have mainly depended on more or less phenomenological approaches, relying on parameters adjusted to limited experimental data or deduced from systematic trends. While these predictions are expected to be reliable for nuclei not too far from experimentally accessible regions, they become questionable when dealing with exotic nuclei. These challenges can be addressed by preferably relying on methods that are as fundamental (microscopic) as possible and based on physically sound models.

Going beyond parametrized analytical formulas, microscopic models of NLD have been developed. When considering publicly available global NLD models that offer predictions for a large number of nuclei, only a limited number of methods are available. Notably, these include the statistical model based on mean-field single-particle scheme and pairing properties [75] and the combinatorial approach [63,76,77]. All these models are based on the independent-particle approximation, and thus, at energies below a few MeV, they all fail to accurately reproduce the detailed, structure-dependent distribution of low-lying levels, especially vibrational ones. Another category of models relies on the configuration interaction approach and employs matrix diagonalization within a physically truncated orbital space [78]. This approach encompasses the shell model and its various adaptations, as well as the moments method, and includes correlations beyond mean-field theory. However, their applications, even within the shell model Monte Carlo method [79] are confined to medium-mass nuclei and can hardly be extended to thousands of nuclei relevant to nuclear applications. The recent QRPA plus boson expansion method [80] also moves beyond the independent particle approximation and exhibits promising predictive power in incorporating low-energy correlations.

All these microscopic methods are rarely used in practical applications for several reasons: (*i*) their limited accuracy in reproducing experimental data, especially when considered globally on a large dataset, (*ii*) their restricted flexibility compared to highly parametrized analytical expressions, or (*iii*) the limited number of nuclei for which NLD calculations have been conducted. The combinatorial approach, as followed in Refs. [63,76], has shown that such models can compete with statistical ones in globally reproducing experimental data. This approach provides energy, spin and parity dependence of NLDs for an extended set of a few thousand nuclei and, at low energies, describes the non-statistical limit, which, by definition, cannot be captured by traditional statistical formulas.

## 4. Impact of PSFs and NLDs on reaction cross sections

The neutron-, proton- or α -capture cross sections and astrophysical rates are calculated here with the TALYS reaction code (v. 1.964) [81]. The photo-reaction rates are obtained from the principle of detailed balance applied to the reverse radiative capture rate. When not available experimentally [82], atomic masses are taken from the BSkG3 mass model [83], the optical model for nucleons from Ref. [84] and the α -channel from Ref. [85]. The other nuclear ingredients, in particular the level scheme and branching ratios are taken from the RIPL-3 database [42]. Since the key nuclear ingredients affecting the calculation of the radiative captures are the NLDs and PSFs, we adopt here seven different combinations based on the following:

*(i*) NLD models,

1. HFB plus combinatorial (HFB+comb) [63],
2. Temperature-dependent HFB plus combinatorial (THFB+comb) [76],
3. Constant-temperature plus Fermi gas (Cst-T) [74],
4. Back-shifted Fermi gas model (BSFG) [74],

and (*ii*) PSF models,

- Gogny-HFB plus QRPA using D1M interaction (D1M+QRPA) [47],
- Simple modified Lorentzian (SMLO) [44],
- Relativistic mean-field + continuum RPA (RMF+cRPA) for the E⁢1 [49] supplemented by the M⁢1 from D1M+QRPA,
- Skyrme-HFB plus QRPA using the BSk27 interaction (BSk27+QRPA) [48].

Note that the above-mentioned models include both phenomenological and microscopic approaches to widely test the associated model uncertainties. Some NLD and PSF models included in the TALYS code were not selected, because either they are considered to be less reliable or redundant with respect to those mentioned above. In particular, the standard [12] or generalized [13] Lorentzian PSF models have been shown to over and underestimate, respectively, the measured average radiative width systematically [44] and a global comparison of NLD models with Oslo method data [64] tends to disfavour the generalized superfluid model [42]. The most tested models recommended by the TALYS authors are models 1 and 4 for NLDs and a and b for PSFs.

The seven combinations of NLDs (from 1 to 4) and PSFs (from a to d) adopted in our sensitivity study are defined as the following: set A (1a), set B (1b), set C (1c), set D (1d), set E (2a), set F (3a) and set G (4a). These combinations all lead to a relatively accurate prediction of the experimental Maxwellian-averaged cross sections (MACS) [86] for 239 nuclei with 20≤Z≤83 . Sets A–D essentially test the impact of PSF models using the same NLDs, while sets A and E–G allow us to study the impact of the NLD models using the same PSF. Note that all de-excitation PSF models (a–d) include a low-energy M⁢1 enhancement (upbend) and a non-zero limit for the E⁢1 [1]. Among the seven combinations, only the D1M+QRPA and SMLO PSFs have been extensively tested on experimental data and are consequently recommended for applications [1]. Note that some popular PSF models, like the standard [87] or generalized [13] Lorentzian models are not (and should not be) considered in this type of sensitivity analysis, since they have been shown to systematically disagree with PSF-related observables such as the average radiative width [47]. Similarly, Cst-T and HFB+Comb NLDs have been largely and satisfactorily used for applications [74,81].

**Figure 1.**
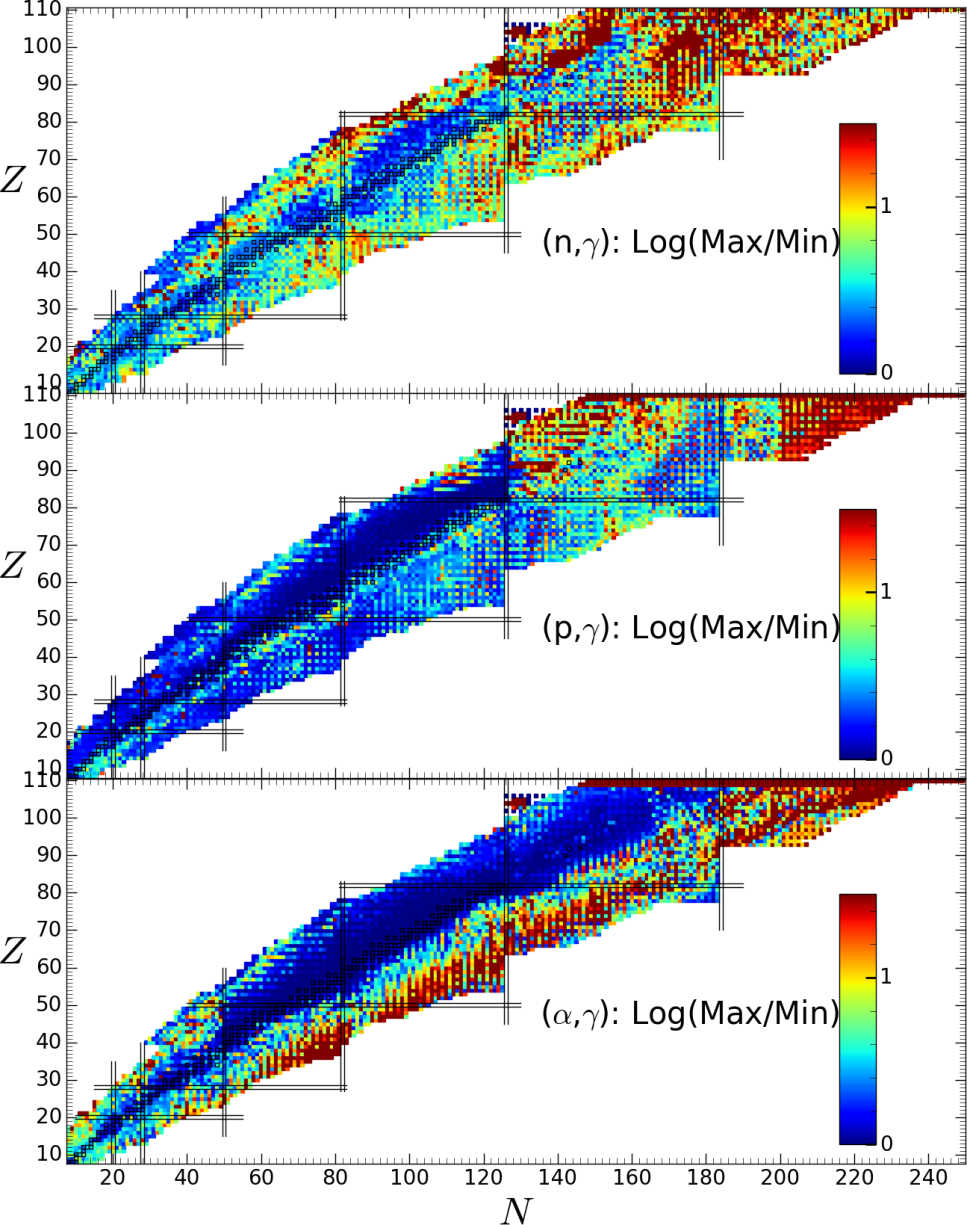
Representation in the ( N , Z ) plane of the ratios between the maximum and minimum astrophysical rates for individual neutron (upper), proton (middle) and α (lower panel) radiative captures obtained with the seven different combinations of NLDs and PSFs in TALYS calculations. The adopted temperature is 2 x $10^{9}$ K.

As illustrated in figure 1, NLDs and PSFs may strongly affect the radiative capture rates, typically up to a factor of approximately 50, especially n- and α -induced reactions on exotic neutron-rich nuclei, as well as heavy nuclei above Pb. However, when dealing with neutron-deficient nuclei, NLDs and PSFs impact mainly the neutron capture, but neither the proton nor the α captures at least at the temperature considered here. The most significant effects responsible for the large impact of the various PSF predictions on the radiative neutron-capture rates are (i) the low-energy E⁢1 strength (including a potential pygmy resonance) predicted for neutron-rich nuclei by mean field plus QRPA approaches and (ii) the low-energy M⁢1 upbend.

## 5. Impact of PSFs and NLDs on nucleosynthesis

### (a) Stellar nucleosynthesis of elements heavier than iron

Astrophysics seeks to address one of the most fundamental questions, delving into the current and past composition of the Universe and its many constituents. Nucleosynthesis theory endeavours to identify the various processes that can be invoked to account for the origin of the observed nuclides in nature, along with pinpointing astrophysical sites capable of fostering the conditions required for these processes to take place [88]. Pivotal contributions to this field can be traced back to the works of Burbidge *et al*. and Cameron [89,90].

The origin of elements lighter than those in the Fe group has primarily been elucidated, thanks to the direct correlation between their nucleosynthesis and the energetic evolution of stars (e.g. [91]). However, the synthesis of nuclei heavier than Fe remains far from well-understood at present. The principal mechanisms invoked to explain the production of heavy nuclei are the s-process [92] occurring during hydrostatic stellar burning phases, the r-process [93,94], thought to manifest during the explosion of a star as a core-collapse supernova (CCSN) or during the coalescence of two neutron stars (NSs) or a NS and a black hole (BH) in a binary system, and the p-process [95,96]), transpiring in CCSNe or Type-Ia supernovae (SNIa). Recently, the i-process [97,98] has also been proposed to explain observed abundances in low-metallicity stars. Since the s-process nucleosynthesis essentially relies on experimental MACS, it will not be discussed here. More information on the i-, r- and p-process nucleosynthesis are given below where only systematic model-correlated uncertainties are propagated and not the parameter (or statistical) uncertainties. It should be noted that, in contrast to many studies, we consider here only PSF and NLD models that cannot be rejected on the basis of existing nuclear data, as well as multi-zone astrophysical simulations that capture the various thermodynamic conditions expected in a given stellar environment. In particular, for the i-, r- and p-processes, sensitivity to nuclear uncertainties should not be tested within a one-zone model. For more details on nuclear uncertainty propagation in astrophysics simulations, the reader is referred to Refs. [99,100].

### (b) Impact of PSFs and NLDs on the i-process

In response to certain observations that could not be fully explained by a combination of the s- and r-processes, a process now termed an intermediate or i-process has been proposed. This i-process is characterized by neutron concentrations in the range of approximately 10^12^ to 10^16^ neutrons/cm^3^. The envisioned mechanism responsible for this production involves the ingestion of protons in convective He- and C-rich layers, resulting in the creation of ^13^C through ^12^C(p, γ )^13^N( $\beta^{+}$ )^13^C, followed by a significant generation of neutrons through ^13^C( α ,n)^16^O. This mechanism shares similarities with the one that invokes to explain the s-process in low-mass asymptotic giant branch (AGB) stars, but the higher neutron concentrations are anticipated owing to the very low metallicity of the star and the activation of ^13^C( α ,n)^16^O in convective regions at high temperatures (typically $∼2.5\times 10^{8}$ K).

Several scenarios have been proposed through numerical simulations to host conditions conducive to the i-process. These include proton ingestion during the core He flash in very low-metallicity low-mass stars, the thermal pulse phase of massive AGB (super-AGB) stars of very low metallicity, the post-AGB phase, rapid accretion of H-rich material on white dwarfs or shell He burning in massive very low-metallicity Population II or III stars. While the contribution of the i-process to the overall Galactic enrichment, and specifically to our Solar System, remains uncertain, it is essential to explain the heavy element patterns in peculiar stars, such as several carbon-enhanced metal-poor (CEMP) stars with the simultaneous presence of s elements and Eu (referred to as CEMP-r/s stars), as well as in Sakurai’s object V4334 Sgr. Further details can be found in Refs. [97,98,100,101] and references therein.

**Figure 2.**
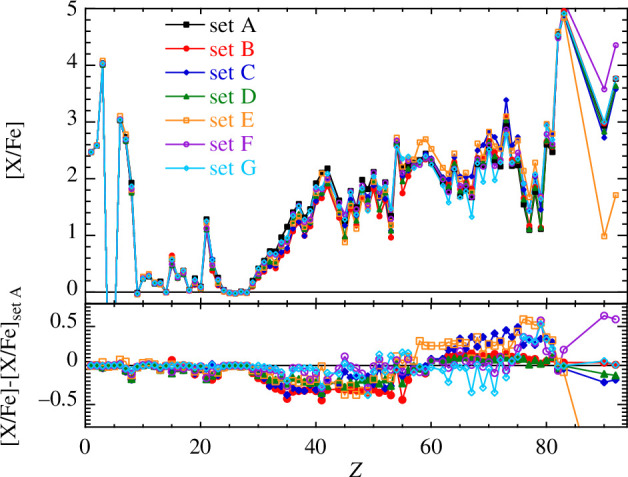
Surface [X/Fe] i-process overabundances in a 1 *M*_⊙_ [Fe/H] = −2.5 AGB model for the seven different nuclear models considered here. The bottom plot shows the differences between the various models and set A. For more information on the i-process model, refer Ref. [100].

The i-process nucleosynthesis essentially involves β -unstable neutron-rich nuclei lying a few nucleons away from the valley of stability for which it remains impossible to experimentally determine neutron-capture cross sections. Since most of the atomic masses are known for such nuclei [82], the key nuclear ingredients affecting the calculation of the radiative neutron captures of relevance to the i-process are the NLDs and PSFs [100]. To illustrate the effect of NLDs and PSFs on the i-process nucleosynthesis, we consider the multi-zone stellar evolution model of the early AGB phase of a low-mass ( $1\,M_{⊙}$ ) low-metallicity ([Fe/H] = −2.5) star, as described in Refs. [98,100]. As shown in figure 2, NLDs and PSFs have a significant impact on the surface abundances of AGB stars subject to the i-process. Deviations of approximately ± 0.5 dex on the overabundance [X/Fe]^1^ are found depending on the adopted model; these are significant in view of the observational precision of approximately 0.1 dex. In the case of the Th and U production, predictions can even differ by approximately 2 dex with a minimum production obtained with THFB+comb NLDs and a maximum with Cst-T NLDs. SMLO and BSk27+QRPA PSFs give almost identical yields. Note that the predicted abundances are correlated by the model and predictions in figure 2 should consequently not be seen as an uncertainty band. In particular, abundance ratios may be more accurately predicted. More details can be found in Ref. [100].

### (c) Impact of PSFs and NLDs on the r-process

The r-process in stellar nucleosynthesis is essential for explaining the production of stable (and some long-lived radioactive) neutron-rich nuclides heavier than iron observed in stars of various metallicities, including in the Solar System. Reviews on this topic are available in Refs. [88,93,94].

Over the years, various nuclear-physics-based and astrophysics-free models of the r-process, varying in sophistication, have been developed. Advances in modelling type-II supernovae and γ -ray bursts have generated excitement around the neutrino-driven wind environment. However, achieving a successful r-process *ab initio* remains elusive without tuning relevant parameters (neutron excess, entropy and expansion timescale) in a manner not supported by the most sophisticated existing models [102]. While these scenarios hold promise, especially with their potential to contribute significantly to the Galactic enrichment, they are plagued by significant uncertainties, primarily stemming from the still incompletely understood mechanism behind supernova explosions and the persistent challenges in obtaining suitable r-process conditions in self-consistent dynamical explosion and NS cooling models [102–104]. In particular, a subclass of CCSNe, known as collapsars and corresponding to the fate of rapidly rotating and highly magnetized massive stars, often considered as the origin of observed long γ -ray bursts, could emerge as a promising r-process site [105–107]. The production of r-nuclides in these events may be associated with jets predicted to accompany the explosion or with the accretion disk forming around a newly born central BH.

Since the early 2000s, special attention has been directed towards NS mergers as potential r-process sites, especially after hydrodynamic simulations confirmed the ejection of a non-negligible amount of matter from the system. Newtonian, conformally flat general relativistic, as well as fully relativistic hydrodynamical simulations involving NS–NS and NS–BH mergers with microphysical equations of state, have shown that typically a fraction, ranging from $10^{-3}\,M_{⊙}$ up to more than 0.1 $M_{⊙}$ , can become gravitationally unbound on roughly dynamical timescales owing to shock acceleration and tidal stripping. Additionally, the relic object, either a hot, transiently stable hyper-massive NS followed by a stable supermassive NS, or a BH-torus system, can lose mass through outflows driven by various mechanisms [108].

Increasingly sophisticated simulations have reinforced the idea that the ejecta from NS mergers serve as a viable r-process sites up to the third abundance peak and the actinides. The enrichment of r-nuclides is predicted to originate from both the dynamical (prompt) material expelled during the NS–NS or NS–BH merger phase and the outflows generated during the post-merger remnant evolution of the relic NS-torus and BH-torus system [108,109]. The resulting abundance distributions align closely with the Solar System distribution and various elemental patterns observed in low-metallicity stars [94]. Moreover, the ejected mass of r-process material, combined with the predicted astrophysical event rate (around 10 Myr^−1^ in the Milky Way), can account for the majority of r-material in our Galaxy. Another compelling piece of evidence supporting NS mergers as r-nuclide producers comes from the highly important gravitational wave and electromagnetic observation of the kilonova GW170817 in 2017 [110,111].

**Figure 3.**
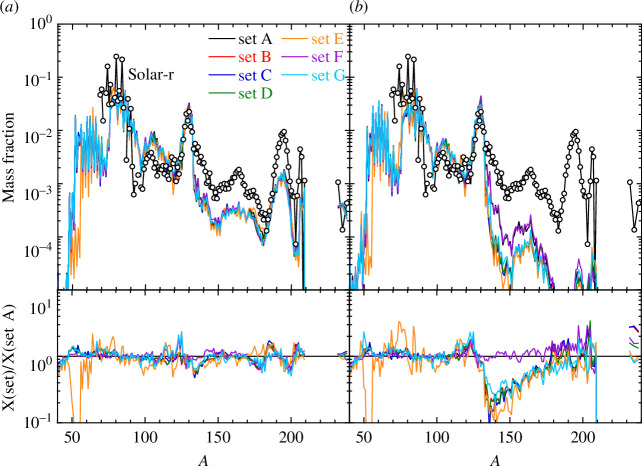
Final r-process mass fractions of stable nuclei (and long-lived Th and U) of the material ejected of a binary 1.38–1.38 *M*_⊙_ NS merger as a function of the atomic mass A obtained with the seven different combinations of NLDs and PSFs for TALYS calculations of all reaction rates. Panel (*a*) shows the full ejecta, panel (*b*) the ejecta along the polar direction ( $|\theta |<45^{\circ }$ ). The solar system r-abundance distribution (open dotted circles) [113] is shown for comparison and arbitrarily normalized. The lower panel shows the mass fraction ratios relative to set A. For more information on the r-process model, refer Ref. [109].

Despite recent successes in nucleosynthesis studies for NS mergers, the details of r-processing in these events is still subject to various uncertainties, particularly in the nuclear physics input (e.g. [99,112]). We illustrate in figure 3 the impact of PSFs and NLDs on the r-process nucleosynthesis in NS merger ejecta corresponding to a binary 1.38–1.38 $M_{⊙}$ NS system [109]. The overall ejecta (*a*) composed of the three dynamical, NS-torus and BH-torus contributions are found to be rather insensitive to the reaction rates owing to the establishment of an (n, γ )–( γ ,n) equilibrium in most of the ejecta. Local deviations can still be observed in particular for 50<∼A<∼70 nuclei when using the THFB+comb NLDs, but also, to a lower extent, around the top of the second and third peaks. Figure 3*b* shows the corresponding part of the ejecta along the polar axis (within the angular region $|\theta |<45^{\circ }$ ), dominated by the NS-torus and BH-torus components. In this case, larger deviations can be observed with a non-negligible NLD impact, in particular, on lanthanide production, hence on a potential observational signal [110]. Also note that owing to the (n, γ )–( γ ,n) equilibrium, in the present model, the inclusion of a PSF low-energy upbend is found to have a negligible impact on the final abundance distribution.

### (d) Impact of PSFs and NLDs on the p-process

The p-process on stellar nucleosynthesis aims to elucidate the production of stable neutron-deficient nuclides heavier than iron observed in the Solar System, and so far, not found in any other Galactic location (for a comprehensive review, refer to Ref. [95]). Various scenarios have been proposed to account for the bulk p-nuclide content of the Solar System. In most of these scenarios, p-isotopes are generated by photo-disintegration reactions acting on previously synthesized s- and r-nuclei. These photo-reactions encompass ( γ ,p), ( γ ,n) and ( γ , α ) reactions at stellar temperatures around 2–3 x $10^{9}$ K.

The p-nuclides are primarily produced during the final explosion of a massive star ( $M\;>\;∼\;10\;M_{⊙}$ ) as a CCSN or during its pre-explosive oxygen burning episode [95]. The p-process can occur in the O–Ne layers of massive stars explosively heated to peak temperatures ranging between 1.7 and $3.3\times 10^{9}$ K. Seeds for the p-process are provided by the s-process that occurs before the explosion in these stellar mass zones. SNIa have also been proposed as potential sites for the p-process; p-process nucleosynthesis, possibly accompanying the deflagration or delayed detonation regimes, has been predominantly studied in one-dimensional simulations, revealing overabundances similar to CCSN models [95,114]. However, predicted p-nuclide yields from SNIa suffer from considerable uncertainties affecting the adopted explosion models and the s-seed distributions, with detailed information on the composition of the material transferred to the white dwarf before the explosion being unavailable.

**Figure 4.**
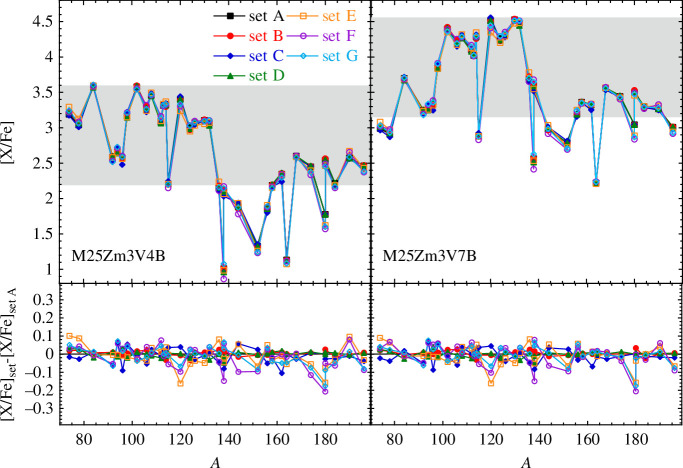
[X/Fe] overabundances in the p-process layers for all the 35 p-nuclides ejected from a 25 *M*_⊙_ model star with an initial rotational velocity $v_{\mathrm{ini}}/v_{\mathrm{crit}}=0.4$ (left panel) or 0.7 (right panel). Abundances are obtained with the seven different combinations of NLDs and PSFs for TALYS calculations of all reaction rates. The grey area shows variation down by an arbitrary factor of 20 with respect to the most overabundant p-nucleus. The bottom plot shows the differences between the various models and set A. For more information on the p-process model, refer Ref. [96].

To illustrate the impact of PSFs and NLDs on p-process nucleosynthesis, we consider exploding rotating massive stars with $25\,M_{⊙}$ and a metallicity of $Z=10^{-3}$ that have undergone an enhanced s-process nucleosynthesis during their life through rotational mixing [96]. Two initial rotation velocities are adopted, namely, $v_{\mathrm{ini}}/v_{\mathrm{crit}}=0.4$ and 0.7, where $v_{\mathrm{crit}}$ is the critical velocity at which the gravitational acceleration is compensated by the centrifugal force. The resulting overproduction factors (with respect to the Sun composition) obtained with the seven different combinations of nuclear inputs are shown in figure 4 for the 35 p-nuclides. The NLDs and PSFs are seen to have a relatively small impact on the overall p-distribution. Indeed, the photodisintegration rates are estimated from the reverse radiative capture rates on the basis of the detailed balance principle and, as shown in figure 1, neutron-deficient nuclei have p- and α -captures rates (hence photo-rates) rather insensitive to NLDs and PSFs. Deviations in the predicted yields essentially come from the more sensitive photo-neutron rates but their impact on the p-process yields remains marginal.

## 6. Constraining nucleosynthesis processes with PSFs and NLDs

Given the theoretical uncertainties stemming from different combinations of NLD and PSF models, it is of utmost importance to provide experimental constraints to obtain more reliable reaction rates for nucleosynthesis simulations. A number of recent results on PSFs and NLDs demonstrate the importance of experimentally constraining reaction rates, thereby enhancing our understanding of nucleosynthesis processes. For completeness, it should be pointed out that the surrogate method is an alternative indirect approach to obtain information on capture cross sections [115,116] but this technique is beyond the scope of this work. A few noteworthy results, based on PSF and NLD measurements, are briefly summarized:

— Measurements of the PSFs and NLDs in ^138,139,140^La have provided experimental data [38] to calculate MACS. From these, ( γ ,n) rates were determined which confirms that the production rate of ^139^La( γ ,n) is smaller than the destruction rate of ^138^La( γ ,n). This implies that ^138^La cannot be produced sufficiently by photo-disintegration during the p-process. The dominant production process for ^138^La remains the electron neutrino capture on ^138^Ba during the p-process in CCSNe [117–119].
— The NLDs and PSFs were measured in ^180,181,182^Ta [17] and used to constrain the calculation of the astrophysical rates of relevance to the nucleosynthesis of ^180^Ta by the s- and p-processes. Nucleosynthesis simulations indicate that the s-process contribution to the Galactic enrichment of ^180^Ta can be neglected and that the primary production mechanism stems from the p-process [120].
— The ^66^Ni(n, γ ) cross section was constrained from PSF and NLD measurements on ^67^Ni using the inverse Oslo Method [20] with radioactive ^66^Ni beams. Simulations demonstrate that the ^66^Ni(n, γ ) reaction acts as a bottleneck when using a simplified one-zone model but its impact on i-process nucleosynthesis is negligible in realistic multi-zone low-metallicity AGB stellar models [16].
— The abundance of the p-nuclide ^92^Mo is typically underestimated in p-process nucleosynthesis calculations. Measurements of the PSF and NLD provided constraints on the ^91^Nb(p, γ )^92^ Mo capture cross section and confirmed that the discrepancy between the ^92^Mo observed and calculated abundances is not attributable to the lack of nuclear physics data [121]. In addition, the relatively low sensitivity of the p-process predictions with respect to uncertainties associated with NLD and PSF implies that solutions to this long-standing problem should be sought in improved astrophysical modelling.

## 7. Conclusion

Nuclear physics is essential to understand stellar evolution, element creation and stellar explosions. PSFs and NLDs affect reaction outcomes in astrophysical settings, shaping elemental abundance patterns through nucleosynthesis processes ranging from radiative capture to photo-disintegration reactions. Advances in theory and experimentation are enhancing our grasp of the origin of elements. A wide range of experimental techniques provide insight into the PSFs and NLDs of nuclei and provide constraints on reaction cross sections and, as such, for nucleosynthesis processes. The impact of several combinations of NLD and PSF models on the i-, r- and p-processes has been investigated. For the i-process, variations in NLDs and PSFs lead to factors of approximately 3–5 deviations in surface abundances for Z > 30 nuclei increasing to approximately two orders of magnitude for actinides. In the case of the r-process, the overall ejecta composition is primarily affected by NLD and PSF model variations for nuclei with A < 60, while in polar ejecta, the abundance distribution is also affected significantly for A > 140 nuclei by up to an order of magnitude. For the p-process, the impact of NLD and PSF models on the yields is found to be relatively small. Overall, these findings emphasize the importance of accurate experimental nuclear data inputs to guide a comprehensive understanding of nucleosynthesis outcomes in various astrophysical environments. As highlighted by recent measurements of PSFs and NLDs such data have proven crucial in constraining models. The impact on improved astrophysical reaction rates and nucleosynthesis based on the availability of experimental nuclear data has been demonstrated, as exemplified by studies of ^67^Ni, ^92^Mo, ^138^La and ^180^Ta. These cases illustrate the contributions PSF and NLD measurements can make in unravelling the nucleosynthesis scenarios taking place in the cosmos.

## Data Availability

Data are generated through the reaction code TALYS, which is freely available for download: https://nds.iaea.org/talys/.

## Footnotes

^1^
The [X/Fe] ratio of an element k is defined as [X/Fe] = log_10_(X_k_/X_Fe_) − log_10_(X_k,⊙_/X_Fe,⊙_), where X_k_ is the elemental mass fraction of the element k and X_k,⊙_ its value in the Solar System.
